# Liposomes loaded with vitamin D3 induce regulatory circuits in human dendritic cells

**DOI:** 10.3389/fimmu.2023.1137538

**Published:** 2023-06-09

**Authors:** Noémi Anna Nagy, Fernando Lozano Vigario, Rinske Sparrius, Toni M. M. van Capel, Ronald van Ree, Sander W. Tas, I. Jolanda M. de Vries, Teunis B. H. Geijtenbeek, Bram Slütter, Esther C. de Jong, for the DC4Balance consortium

**Affiliations:** ^1^ Amsterdam Universitair Medische Centra (UMC), Department of Experimental Immunology, Amsterdam Institute for Infection & Immunity, University of Amsterdam, Amsterdam, Netherlands; ^2^ Division of BioTherapeutics, Leiden Academic Center for Drug Research, Leiden, Netherlands; ^3^ Amsterdam Universitair Medische Centra (UMC), Department of Otorhinolaryngology, University of Amsterdam, Amsterdam, Netherlands; ^4^ Amsterdam Universitair Medische Centra (UMC), Department of Rheumatology and Clinical Immunology, University of Amsterdam, Amsterdam, Netherlands; ^5^ Department of Tumor Immunology, Institute for Molecular Life Sciences, Radboud University Medical Center, Nijmegen, Netherlands

**Keywords:** liposome, vitamin D3, dendritic cells, T regulatory cells (Tregs), immunotherapy, tolerance, autoimmunity, allergies

## Abstract

**Introduction:**

Nanomedicine provides a promising platform for manipulating dendritic cells (DCs) and the ensuing adaptive immune response. For the induction of regulatory responses, DCs can be targeted *in vivo* with nanoparticles incorporating tolerogenic adjuvants and auto-antigens or allergens.

**Methods:**

Here, we investigated the tolerogenic effect of different liposome formulations loaded with vitamin D3 (VD3). We extensively phenotyped monocyte-derived DCs (moDCs) and skin DCs and assessed DC-induced regulatory CD4+ T cells in coculture.

**Results:**

Liposomal VD3 primed-moDCs induced the development of regulatory CD4+ T cells (Tregs) that inhibited bystander memory T cell proliferation. Induced Tregs were of the FoxP3+ CD127low phenotype, also expressing TIGIT. Additionally, liposome-VD3 primed moDCs inhibited the development of T helper 1 (Th1) and T helper 17 (Th17) cells. Skin injection of VD3 liposomes selectively stimulated the migration of CD14+ skin DCs.

**Discussion:**

These results suggest that nanoparticulate VD3 is a tolerogenic tool for DC-mediated induction of regulatory T cell responses.

## Introduction

With a worldwide rise in prevalence of both allergic and autoimmune conditions, the need for developing specific and efficient tolerizing immunotherapies is more relevant than ever (1). To date, the only tolerizing treatment with curative potential is allergen immunotherapy (AIT), but some well-known disadvantages afflict it. Sustained efficacy of AIT is dependent on at least 3 years of monthly injections that carry the risk of potentially life-threatening side effects, both factors negatively affecting patient adherence (2–4). In contrast to allergy treatment, no curative options exist for autoimmune conditions, and more specific alternatives to broadly immune suppressive therapies are required.

Dendritic cells (DCs) are immune cells under scrutiny for dictating a therapeutic tolerogenic response, as they can foster peripheral tolerance through promoting deletion of effector T cells and the induction of regulatory T cells (Tregs) (5–7). DCs can be manipulated by tolerogenic adjuvants to induce immune regulation. Several forms of vitamin D, including the active vitamin D metabolite 1,25 α-dihydroxy vitamin D3 (VD3), are endowed with pluripotent immunosuppressive activity, and clinical evidence suggests beneficial effects in rheumatoid arthritis, psoriasis, or as an additive to allergen immunotherapy (8–10). VD3 can directly stimulate forkhead box protein (FoxP3)+ Treg development (11) or mediates tolerogenic effects through the interaction with DCs (12–15). VD3 inhibits maturation of and IL-12 production by DCs but also induces the expression of tolerogenic molecules and cytokines, such as Ig-like transcript 3 (ILT3), as well as IL-10 (16, 17). Most importantly, VD3-treated DCs demonstrate exceptional resistance to proinflammatory stimulation after repeated rechallenge, making VD3 a robust tolerance-promoting adjuvant (8).

Given the crucial role of DCs in immune tolerance, several phase I clinical trials are now applying *ex vivo* DC therapy with an ultimate curative aim for rheumatoid arthritis and multiple sclerosis, using, amongst other components, VD3 to create tolerogenic DCs (18–22). However, *ex vivo* DC therapy is costly and requires personalized application (23). A different approach is targeting DCs *in vivo* using a tolerogenic vaccine formulation, passing the necessity of personalization. Nanoparticles could be of aid in this approach as they offer the possibility to unite adjuvant, disease-relevant antigen, and cell-specific targeting molecules in one spatial unit (24, 25). In addition, nanoparticles protect their content from degradation and causing harmful effects in bystander cells. This advantage is relevant for a compound such as VD3, given its instability and toxic effects in high doses (10, 26). Liposomes are biocompatible nanoparticles with a lipid bilayer, adjustable in size, rigidity, and surface electric charge with relative ease (25). For this study, we selected an anionic and a cationic formulation with similar size and rigidity from a larger array of liposomal formulations that we previously evaluated as putative tolerogenic vaccine carriers (27).

One readily accessible site for delivery of a liposomal vaccine is the skin, where several subsets of DCs reside. Due to continuous exposure to harmless bacterial flora, Langerhans cells (LCs) in the epidermis and the dermal CD1a+ or CD14+ skin DC subsets are equipped with efficient tolerogenic properties in steady state (28, 29), and can be targeted with intradermal injection of liposomes (30).

It is currently unknown how VD3-loaded liposomes affect DCs and the ensuing adaptive T cell response. Therefore, to deliver *in vitro* proof of concept for a DC-targeted tolerogenic nanoparticle therapy, we investigated the effects of VD3-loaded liposomal formulations on monocyte-derived DCs (moDCs) and the ensuing T cell response. Additionally, we applied an *ex vivo* human skin model to examine the effect of liposomal VD3 injection on skin DC crawl-outs. We demonstrate that liposomal VD3 efficiently induces tolerogenic DCs that promote the outgrowth of functional Tregs and suppress T helper 1 (Th1) and T helper 17 (Th17) responses. When injected in *ex vivo* human skin, VD3-loaded liposomes selectively enhance the migration of CD14+ DDCs, suggesting ongoing tolerogenic processes *in situ*. Taken together, these data demonstrate proof-of-concept for the efficacy of VD3-loaded liposomes in tolerizing DCs, resulting in the regulation of T cell responses.

## Materials and methods

### Liposome preparation

Anionic liposomes containing 1,2-distearoyl-sn-glycero-3-phosphoglycerol (DSPG) and cationic liposomes containing 1,2-dipalmitoyl-3-trimethylammonium-propane (DPTAP) (**Table 1**) were manufactured using the thin film dehydration-rehydration method, as described elsewhere (27, 31, 32). For the vitamin-loaded formulations, 150 μg VD3 (Sigma Aldrich, St Louis, Missouri) dissolved in ethanol was added to approximately 3 mg of lipids in the lipid mix. VD3-loaded liposomes were dialyzed overnight using a Spectra-Por^®^ Float-A-lyzer^®^ dialysis kit (MWCO 100,000 Da) against 400 ml 10 mM phosphate buffer (PB) pH 7.4 to separate non-encapsulated VD3. Liposomes were stored at 4 °C in PB and used for further experiments within 3 months.

**Table 1:** Physicochemical properties of VD3-loaded liposomal formulations.

Formulation	Lipid composition	Z-average (nm)	± SD	PdI	± SD	ζ potential (mV)	± SD	LE (%)	± SD
**DSPG(-) VD3**	DSPC : DSPG : CHOL	183	12.63	0.14	0.07	-39.5	11.6	1.86	0.02
**DSPG(-) VD3**	DOPC : DSPG : CHOL	168	11.55	0.16	0.04	-46.33	4.92	63	3.65
**DPTAP(+) VD3**	DSPC : DPTAP : CHOL	205	28.9	0.13	0.03	29.0	2.33	2.37	3.30
**DPTAP(+) VD3**	DOPC : DPTAP : CHOL	184	11.60	0.19	0.04	25.9	2.64	62	2.69

Characteristics are shown as mean ± SD of n=3 different batches. DSPC, 1,2-distearoyl-sn-glycero-3-phosphocholine. DOPC, 1,2-dioleoyl-sn-glycero-3-phosphocholine. LE, loading efficiency of VD3.

### Quality control of liposomes

Quality control was performed as previously described (27). For stability testing, measurements were repeated each month after liposome preparation (**Table 2**). To confirm lipid concentration of the formulations and VD3 concentration encapsulated in the liposomes, reversed-phase ultra-performance liquid chromatography (Waters ACQUITY UPLC, Waters, Massachusetts) was used, as described (27). VD3 was detected by absorbance at 252 nm using an ACQUITY UPLC TUV detector (Waters). Loading efficiency (LE) of VD3 was calculated as

**Table 2:** Stability measurements of DSPG and DPTAP VD3-loaded liposomal formulations.

DSPG(-) VD3						
time (months)	Z-ave (nm)	± SD	PdI	± SD	ζ-potential (mV)	± SD
0	168	9.78	0.16	0.03	-48.33	5.57
1	169	11.40	0.14	0.03	-40.49	5.78
2	180	6.81	0.16	0.03	-42.30	3.85
3	170	7.20	0.18	0.01	-49.29	5.41
DPTAP(+) VD3						
time (months)	Z-ave (nm)	± SD	PdI	± SD	ζ-potential (mV)	± SD
0	180	9.69	0.18	0.04	27.01	3.00
1	183	15.11	0.19	0.03	27.46	4.45
2	197	13.23	0.20	0.02	26.90	5.02
3	175	19.70	0.18	0.04	28.52	2.62

Stability was monitored over the course of 3 months using Z-ave, PdI, and ζ-potential measurements. Measurements are expressed as mean ± SD of n=3-5 liposome batches.


$$LE\;\left(\%\right)=\frac{VD3\;concentration\;after\;dialysis}{VD3\;concentration\;before\;extrusion}\;*100.$$

As the loading efficiency of VD3 proved marginal with the head lipid DSPC in the formulations, the head lipid was replaced by DOPC, leading to markedly improved loading efficiency (**Table 1**). All formulations had a size of less than 250 nm and a PdI of less than 0.2, indicating a monodisperse quality (**Table 1**). Measured ζ-potential corresponded to the expected surface charge of the formulations. Formulations were stable throughout their use (**Table 2**).

### *In vitro* generation and activation of moDCs

MoDCs were differentiated from peripheral blood monocytes obtained from buffy coats or fresh blood as described elsewhere (33). Briefly, monocytes were isolated from peripheral blood mononuclear cells (PBMCs) *via* density centrifugation and subsequently cultured for 5-7 days in Iscove’s Modified Dulbecco’s Medium (IMDM, Gibco, Paisley, UK) supplemented with gentamicin (86 μg/ml; Duchefa, Haarlem, The Netherlands), 5 % fetal calf serum (FCS) (Gibco), granulocyte-macrophage colony-stimulating factor (GM-CSF, 500 U/ml; Schering-Plough, Uden, The Netherlands) and IL-4 (10 IU/ml; Miltenyi Biotech, Bergisch Gladbach, Germany). Healthy volunteers for blood sampling were recruited per the Academic Medical Center Medical Ethical Committee (protocol nr. 2015_074). For assessment of moDC surface markers, cells were matured for 48 hours with IL-1β (25 ng/ml) and TNF-α (50 ng/ml) (both purchased from PBH, Hannover, Germany; this combination will be referred to as maturation factor or ‘MF’) and *Escherichia coli* lipopolysaccharide (LPS) with or without liposomal or soluble VD3 (0.01-2.5 μM) and with or without empty liposomes DSPG or DPTAP as control. Lipid concentration in empty liposome conditions was adjusted to the lipid concentration of VD3-loaded liposomes used in the experiments. As each batch of liposomes differed in LE of VD3, lipid concentrations of empty batches used in the experiments had to be adjusted accordingly. For extensive assessment of co-stimulatory and co-inhibitory markers, moDCs were surface stained with anti-HLA-DR-BV421, anti-ILT2 (CD85J)-BV480, anti-ILT3 (CD85k)-BV510, anti-B7H3 (CD276)- BV750, anti-CD86-FITC, anti-ICOSL-PE-CF594, anti-CD83-PE-Cy5 (all BD Biosciences), and anti-ILT4 (CD85d)-PE, as well as anti-PD-L1 (CD274)-PE-Cy7 (all eBioscience, Thermo Fisher) and 10.000 DCs (3) acquired on the SP6800 Spectral Analyzer (Sony).

### Isolation of naïve and memory CD4+ T cells

The total CD4+ T-cell population was isolated from PBMCs by negative magnetic selection using the MACS CD4+ T cell isolation kit (Miltenyi Biotech). Subsequently, naïve CD4+CD45RA+ cells were purified by negative selection, and CD4+CD45RO+ memory cells by positive selection using anti-PE beads (Miltenyi Biotech). Purity of isolated populations exceeded 95 % and was analyzed by flow cytometry using anti-CD4-APC, anti-CD45RA-FITC (all BD), and anti-CD45RO-PE (DAKO, Agilent, Santa Clara, California).

### Stimulation and analysis of CD4+ T cells

For phenotypic analysis, 20.000 allogeneic naïve CD4+ T-cells were stimulated in 200 μl IMDM with 10 % FCS with 10 pg/ml *Staphylococcus aureus* enterotoxin B (Sigma-Aldrich) as described (33), and 5000 MF+LPS-activated DCs that were previously matured for 48 hours with or without liposomal or soluble VD3. DCs were washed three times with 3 ml medium prior to use in culture, given that VD3 has been demonstrated to have a profound direct effect on T cells (34, 35). On day 5 of the coculture, effector T cells were gently harvested and incubated in a new culture plate with human rIL-2 (10 U/ml, Cetus, Emeryville, CA), leaving moDCs attached in the wells of the original culture plate. When resting (coculture day 10-12), a maximum of 500.000 effector T cells was restimulated for 5 hours with phorbol 12-myristate 13-acetate (100ng/ml)/ionomycin (1 μg/ml, Sigma) + brefeldin (10 μg/ml, Sigma) followed by intracellular staining for IFN-γ-FITC (BD) and IL-13-PE (BD), as described previously (33, 36). Additionally, 100.000 CD4+ T cells were stimulated with soluble murine mAb to human CD3 (0.5 μg/mL; Sanquin Research, Amsterdam, The Netherlands) and CD28 (1 μg/mL, Sanquin Research) in 200 μL of IMDM for analysis of IL-10 in 24-hour supernatants. To phenotype Tregs, resting T cells were stained for CD25, CD127, FoxP3, or with a panel of antibodies for CD39, CD49b, CD69, programmed cell death 1 (PD-1), T-cell immunoglobulin and ITIM domain (TIGIT), T-cell immunoglobulin and mucin domain 3 (TIM-3), CD127, CD25, inducible-co-stimulator (ICOS), cytotoxic T-lymphocyte associated protein (CTLA-4), FoxP3, and LAG-3 as described elsewhere (37). Cells were acquired in the live gate on a FACS Canto A (BD) or the SP6800 Spectral Analyzer (Sony). For suppressor assays, 300.000 allogeneic naïve CD4+ T cells were cocultured for 6 days with 30.000 MF+ LPS-activated, soluble, or liposomal VD3-treated moDCs. After 6 days of coculture with moDCs, the emerging effector T cells were gently harvested, and irradiated at 30 Gray to prevent further expansion.. 50.000 effector T cells were subsequently used with 25.000 memory CD4+ T cells labeled with carboxyfluorescein diacetate succinimidyl ester (CFSE) (0.5 mM; Molecular Probes, Eugene, Oregon) as target cells (36).

### Autologous coculture of naïve CD4+ T cells with moDCs and neutrophils

On day 6 of DC generation, immature DCs were primed for 2 hours with 2.5 µM VD3 or 2.5 µM VD3-containing DSPG liposomes before harvesting. Cocultures with DCs, autologous neutrophils, and CD4+ T cells were done in *C. albicans* hyphae-coated plates in IMDM medium containing 5 % human serum, as described elsewhere (37–39). Neutrophils were isolated fresh on the first day of coculture, from the erythrocyte pellet of Lymphoprep density centrifugation, as described (39). The pellet was lysed for 15 minutes on ice with 0.155 mol NH4CL (Sigma-Aldrich), 1mM KHCO3 (Merck, Darmstadt, Germany), and 80 μM EDTA (Merck) dissolved in pH 7.3 sterile water. After centrifugation and repeated lysis of 5 minutes, neutrophils were resuspended in IMDM 5 % human serum.

### Extraction and priming of skin DCs

*Ex vivo* DCs were obtained from healthy human skin as described elsewhere (40). Intradermal injections were carried out with 50 μl of phosphate-buffered saline, LPS+MF, VD3 (25 μM), and liposomes DSPG or DPTAP with or without VD3 in concentrations corresponding to the soluble vitamin control. Migrating cells were stained for the skin DC markers with anti-CD11c-PE-Cy7 (eBioscience, Thermo Fisher), anti-HLA-DR-PercP (BD), anti-CD1a-FITC (BD), and anti-CD14-APC-Cy7(BD).

### Data analysis and statistics

Flow cytometric data were analyzed using FlowJo™ Software (for Windows, Version 10.6.2., Ashland). Heatmaps were generated using Tercen™. Statistical analyses were conducted using GraphPad Prism software (GraphPad, La Jolla, CA).

## Results

### Priming of DCs with VD3-loaded liposomes induces CD4+ T cells with regulatory function

**Figure 1:**
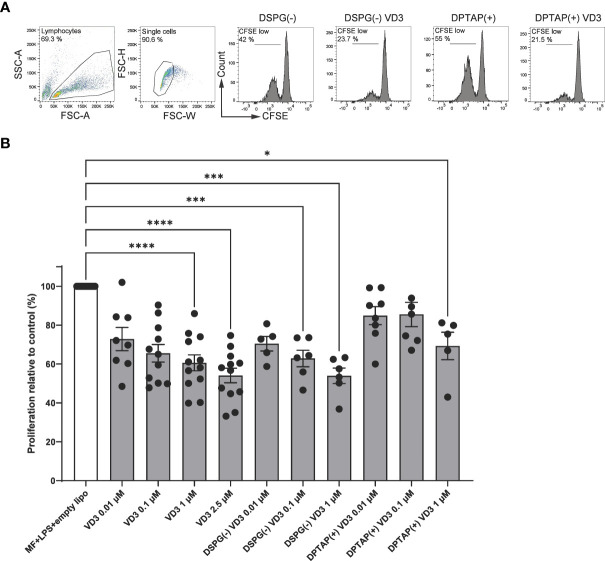
VD3-liposome-treated DCs induce functional Tregs. Allogeneic naïve CD4+ T cells were cocultured with MF+LPS-activated DCs, or DCs activated with MF+LPS and soluble VD3 or VD3-loaded liposomes for 6 days and subsequently used as T cells in coculture with CFSE-labeled CD4+ T memory cells. After 6 days of coculture, proliferation of CD4+ T memory cells was measured with flow cytometry. **(A)** Example gating of CD4+ T memory proliferation after coculture with MF+LPS and liposome DC-primed or MF+LPS and VD3-liposome DC-primed T cells (from left to right). **(B)** CD4+ bystander T memory proliferation normalized to the proliferation induced by MF+LPS and empty DSPG and DPTAP liposome DC-primed T cells, which was set to 100%. Lipid concentrations of empty DSPG batches ranged from 1-2.6 μg/ml, 10-26 μg/ml, 50-260 μg/ml, and of empty DPTAP batches 0.5-1 μg/ml, 2.5-15 μg/ml, 14-40 μg/ml, adjusted to the lipid concentration added when using 0.01, 0.1 or 1 μM liposome-incorporated VD3, respectively. N=5-12 independent experiments. Mean ± SD of proliferation in the control condition was 36% ± 12.4%. Error bars indicate mean ± SEM. *p≤ 0.05. ***p≤ 0.001. ****p≤ 0.0001. Statistical significance was calculated using a mixed-effects model of One-way ANOVA, with Dunnett’s correction for multiple comparisons.

To investigate whether VD3-liposome primed DCs induce functional Tregs, we primed naïve CD4+ T cells with moDCs exposed to VD3-containing DSPG or DPTAP liposomes and determined regulatory T cell capacity in a T-cell suppressor assay. Allogeneic naïve CD4+ T cells were cocultured with VD3 or VD3-liposome primed moDCs and subsequently cocultured with CFSE-labeled CD4+ T memory cells (**Figure 1A**). The maximum concentration of liposome-incorporated VD3 in the suppressor assays was 1 μM, as in the liposome batches used for these experiments, loading efficiency of VD3 was less than 3 % (**Table 1**). T cells primed by VD3-exposed DCs suppressed the proliferation of bystander T cells in a dose-dependent fashion, most pronounced at 2.5 μM of VD3 (**Figure 1B**). Interestingly, DCs primed with either negatively charged DSPG-VD3 or positively charged DPTAP-VD3 induced suppressive Tregs (**Figures 1A, B**). At a VD3 concentration of 1 μM, DCs primed by negatively charged DSPG-VD3 liposomes induced suppressive T cells that inhibited bystander T cell proliferation to similar levels as soluble VD3 at 2.5 μM. DCs treated with positively charged DPTAP-VD3 liposomes also induced suppressive T cells, but to levels seen with 0.01-0.1 μM soluble VD3. In conclusion, our data indicate that DCs primed with 1 μM VD3-loaded liposomes induce functional Tregs, irrespective of liposome type, comparable to soluble VD3-treated DCs.

### DCs activated in the presence of liposomal VD3 induce both FoxP3- and IL-10-expressing Tregs

**Figure 2:**
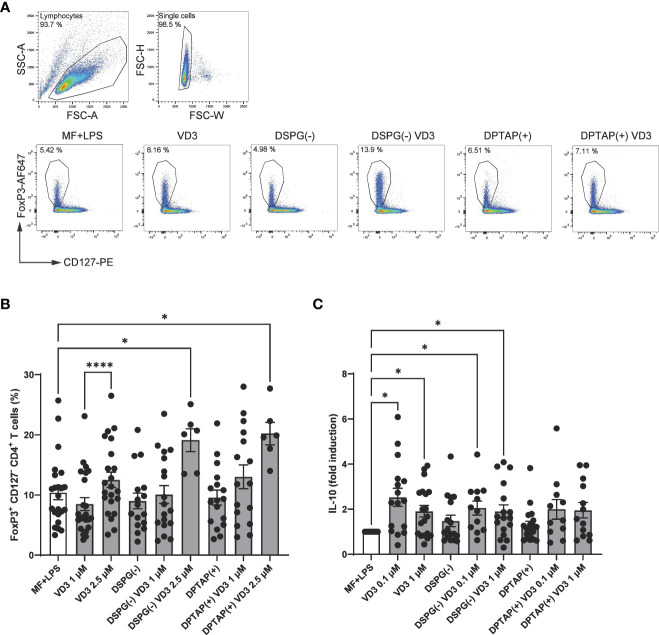
VD3-liposome treated DCs prime for the development of FoxP3+ CD127- CD4+ T cells and IL-10-producing CD4+ T cells. Allogeneic naïve CD4+ T cells were cocultured with MF+LPS activated, VD3 or VD3-liposome primed moDCs for 10-12 days, and frequencies of FoxP3+ CD25+ CD127low cells were measured by FACS. For IL-10 measurement with ELISA, CD4+ T cells were re-stimulated with aCD3, aCD28. **(A)** FoxP3+ CD127low T cells were gated from the single-cell gate. Example dot plots of gating FoxP3+ CD127low T cells and frequencies in example conditions are shown. **(B)** Frequency of FoxP3+ CD127low T cells after stimulation with differently primed moDCs. N=6-23 independent experiments. **(C)** IL-10 production by cocultured T cells after overnight stimulation with aCD3, aCD28 normalized to MF+LPS DC condition, which was set to 1. Lipid concentration of empty DSPG batches shown ranges from 50-260 μg/ml and of empty DPTAP batches 14-86 μg/ml, adjusted to the lipid concentration added when using 1-2.5 μM liposome-incorporated VD3. Mean ± SD of LPS-stimulated IL-10 production in the control condition was 289 pg/ml ± 333 pg/ml. N=11-19 independent experiments. Error bars indicate mean ± SEM. *p≤ 0.05. ****p≤ 0.0001. Statistical significance was calculated using a mixed-effects model of One-way ANOVA, with Dunnett’s correction for multiple comparisons.

As Tregs consist of several subsets, we analyzed the phenotype of functional Tregs induced by liposomal VD3-primed DCs. We first determined the presence of two heterogeneous T cell populations associated with Tregs in human, FoxP3+ CD25+ CD127low CD4+ T cells, as well as IL-10-producing CD4+ T cells. Frequencies of FoxP3+ CD127low CD4+ T cells were strongly increased in coculture with DCs activated in the presence of anionic DSPG liposomes containing 2.5 μM VD3 (**Figures 2A, B**). After treatment with DPTAP liposomes containing 2.5 μM VD3, DCs also induced FoxP3+ CD127low cells. Stimulation of DCs with a lower concentration of 1 μM liposomal VD3 or with soluble VD3 did not result in significant induction of Foxp3+ CD127low CD4+ T cells.

Foxp3+ CD4+ T cells also rely on IL-10 production as a means of suppressing inflammation, together with type 1 regulatory T cells (Tr1), which have been shown to be induced by VD3-treated DCs (41). IL-10 production was increased in T cells primed by DCs treated with 0.1 μM VD3 (**Figure 2C**). Interestingly, only DCs primed with VD3-containing DSPG liposomes significantly stimulated IL-10 production compared to the control condition, while with DPTAP-VD3 primed moDCs, only a trend of IL-10 induction was visible. Our data suggest that VD3-liposome-treated moDCs at a concentration of 2.5 μM VD3 induce FoxP3+ T cells in coculture, while IL-10 producing T cells are significantly fostered by 0.1-1 μM VD3-stimulated or 0.1-1 μM VD3-containing DSPG liposome stimulated moDCs.

### A heterogeneous marker profile is induced in FoxP3+ and FoxP3- CD4+ T cells by VD3-liposome-treated DCs

**Figure 3:**
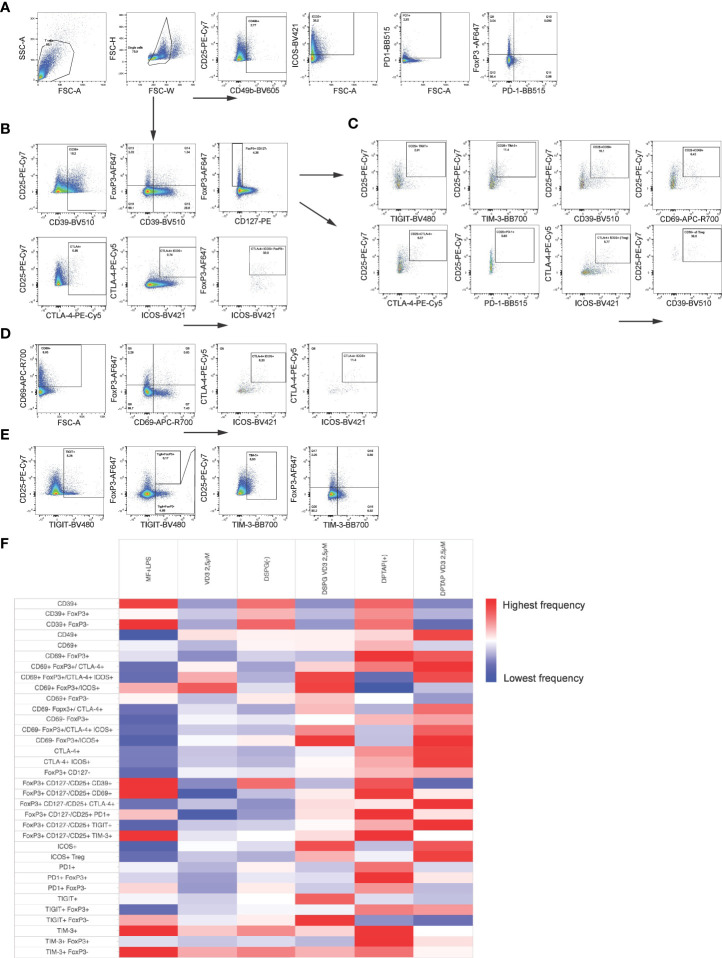
Phenotypic analysis of Tregs induced by VD3-liposome treated DCs. After 10-12 days coculture with differently primed moDCs, CD4+ T cells were stained for Treg subset and functional markers. Arrows indicate which parent population the subpopulation of cells was derived from. **(A)** CD4+ T cells expressing CD49b, ICOS or PD-1 were gated from the single cell gate. PD-1 and FoxP3+ coexpressing cells were gated, as shown in the right panel. **(B)** CD4+ T cells expressing CD39, CD25, CTLA-4, or ICOS were also derived from the single-cell gate. FoxP3+ cells were further examined for CD39 coexpression and CD127 expression. CTLA-4+ cells were gated together with ICOS, and FoxP3+ cells were determined within the double-+ population. **(C)** Example gating for assessing expression of TIGIT, TIM-3, CD39, CD69, CTLA-4, PD-1 and ICOS within the FoxP3+ CD127low CD25+ population of CD4+ T cells. CTLA-4 ICOS coexpressing cells within this population were identified as iTregs and further examined for CD39 expression. **(D)** After gating CD69+ cells from single CD4+ T cells, CD69 expression against FoxP3 expression was assessed with a quadrant gate, and CTLA-4 ICOS coexpression determined within both CD69- (Q5) and CD69+ (Q6) FoxP3+ cells. **(E)** TIGIT and TIM-3 expressing cells were gated from the single cell population of CD4+ T cells and assessed for FoxP3 expression as shown. **(F)** Heatmap representing frequency of indicated T cell populations per DC-activation condition is shown.

**Figure 4:**
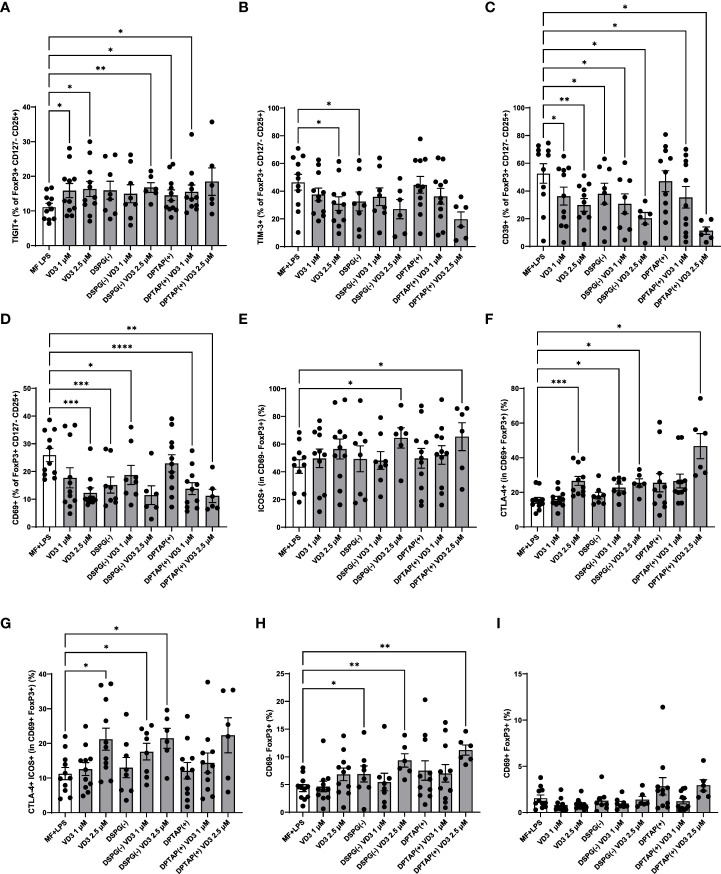
VD3-liposome-treated DCs induce Tregs with a distinct phenotype. **(A)** Frequency of TIGIT+ or **(B)** TIM-3+ cells within the FoxP3+ CD127- CD25+ population of T cells. **(C)** Frequencies of CD39+ cells within FoxP3+ CD127- CD25+ cells. **(D)** Frequencies of CD69+ cells within FoxP3+ CD127- CD25+ cells. **(E)** Frequencies of ICOS+ cells within the CD69- FoxP3+ cell population.**(F)** Frequencies of CTLA-4+ cells within the CD69+ FoxP3+ population. **(G)** Frequencies of CTLA-4+ ICOS+ cells within CD69+ FoxP3+ T cells. **(H)** Frequencies of CD69- FoxP3+ T cells. **(I)** Frequencies of CD69+ FoxP3+ T cells. Lipid concentration of empty DSPG batches shown ranges from 50-260 μg/ml and of empty DPTAP batches 14-86 μg/ml, adjusted to the lipid concentration added when using 1-2.5 μM liposome-incorporated VD3. N=5-11 independent experiments. Error bars indicate mean ± SEM. *p≤ 0.05. **p≤ 0.01. ***p≤ 0.001. ****p≤ 0.0001. Statistical significance was calculated using a mixed-effects model of One-way ANOVA, with Dunnett’s correction for multiple comparisons.

As we observed both induction of FoxP3+ CD127- T cells and IL-10 producers, we phenotyped these heterogeneous cell populations with a flow cytometry panel of different T cell activation and regulatory markers (**Figures 3A-E**). The expression profiles are displayed in a heatmap (**Figure 3F**), indicating enhanced expression of CTLA-4 and TIGIT within the FoxP3+ population of CD4+ T cells, as well as an increased expression of ICOS+ CTLA-4+ FoxP3+ T cells (ICOS+ Tregs) in VD3-DC primed conditions. Priming of DCs with liposomal VD3 led to significantly higher frequencies of cells expressing the co-inhibitory receptor TIGIT within FoxP3+ CD127low CD25+ T cells and the FoxP3+ bulk population (**Figure 4A**, **Supplementary Figure 1A**), compared to the activated DC control condition. In contrast, in the same population, no significant changes in CTLA-4+ cells were observed (**Supplementary Figure 1B**). Even though the co-inhibitory receptor TIM-3, the ectonucleotidase CD39, and the early T cell activation marker CD69 have been described as functional Treg markers (42, 43), these markers were significantly decreased in VD3-liposome treated conditions (**Figure 3F**, **Figures 4B, C, D**). Although the VD3-DC-induced expression changes in ICOS+ Tregs were not significant (**Supplementary Figure 1C**), cells expressing ICOS increased in frequency within the resting CD69- FoxP3+ T cell population (**Figure 3F**, **Figure 4E**). CTLA-4+ cells and CTLA-4 ICOS coexpressing cells, on the other hand, were only increased in the activated CD69+ FoxP3+ T cell population upon coculture with VD3-treated DCs (**Figures 4F, G** **)**. Interestingly, non-activated CD69- FoxP3+ T cells were significantly induced by VD3-liposome-treated DCs but not CD69 coexpressing FoxP3+ T cells (**Figures 4H, I**), suggesting that the VD3-DC stimulated increase in FoxP3+ Tregs is not due to an increase in activated effector T cells.. In VD3-DC instructed FoxP3- T cells, we also observed reduced frequencies of TIM-3, CD39, or PD-1+ cells (**Figure 3F**, **Supplementary Figures 1D-F**). We examined the expression of LAG-3 and CD49b, two markers considered essential for defining Tr1 cells. However, we could not measure reliable changes in this population due to low expression levels of the most critical marker, LAG-3 (data not shown). Even though CD49b appeared differentially expressed in the VD3-DC primed conditions (**Figure 3F**), this change was not significant (**Supplementary Figure 1G**). Taken together, compared to activated DCs, DCs primed with liposomal VD3 induced FoxP3+ CD127- T cells with enhanced expression of TIGIT, while we could not demonstrate induction of FoxP3- Tr1 cells. Interestingly, several functional Treg markers were decreased in soluble and liposomal VD3-primed T cells.

### VD3-liposome primed DCs influence directionality of CD4+ T cell polarization

**Figure 5:**
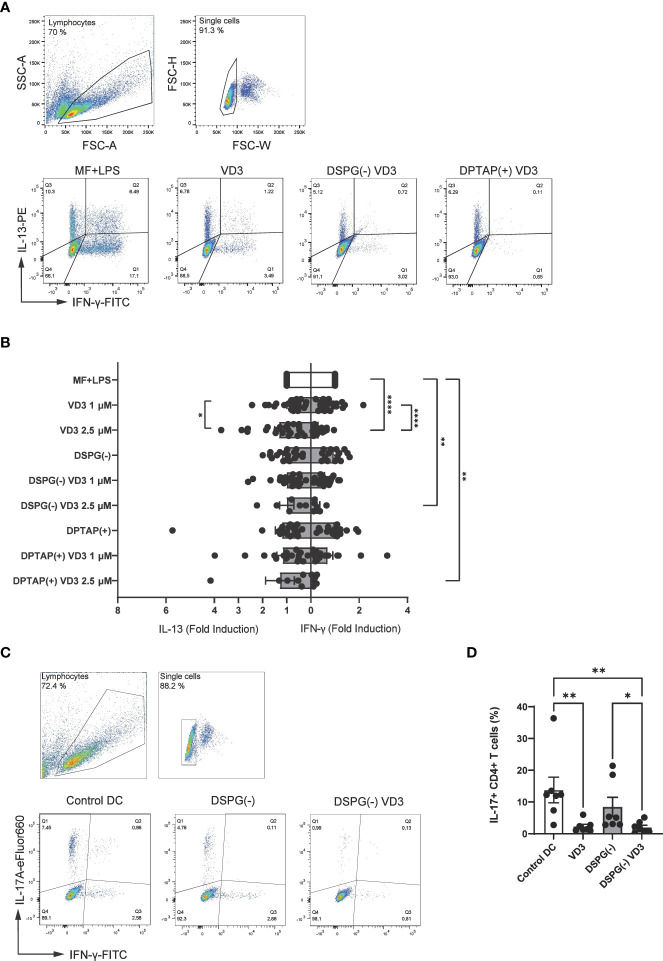
VD3-liposome-treated DCs reduce Th1 and Th17 polarization. **(A)** Example dot plots of gating IFN-γ+ and IL-13+ CD4+ T cells from the single-cell population in MF+LPS DC or VD3-stimulated DC conditions. Allogeneic naïve CD4+ T cells were cocultured with MF+LPS activated, VD3 or VD3-liposome primed moDCs for 10-12 days and stained for IFN-γ+ and IL-13+ after 5-hour stimulation with PMA+Ionomycin. **(B)** Fold-induction of IL-13+ and IFN- γ+ CD4+ T cells in different moDC-priming conditions. Lipid concentration of empty DSPG batches shown ranges from 50-260 μg/ml and of empty DPTAP batches 14-86 μg/ml, adjusted to the lipid concentration added when using 1-2.5 μM liposome-incorporated VD3. Mean ± SD of IFN-γ+ T cells stimulated by MF+LPS DCs was 22.5 % ± 8.9 %, while of IL-13+ T cells, 10.68 % ± 7.34 %. N= 6-23 independent experiments. Error bars indicate mean ± SEM. *p≤ 0.05. **p≤ 0.01. ****p≤ 0.0001. Statistical significance was calculated using a mixed-effects model of One-way ANOVA, with Dunnett’s correction for multiple comparisons. **(C)** Example gating of IL-17 and IFN-γ expressing CD4+ T cells in different priming conditions. **(D)** Frequencies of IL-17+ CD4+ T cells after autologous coculture with neutrophils and differently primed moDCs. Soluble and liposome-incorporated VD3 concentration was 2.5 μM. N=7 independent experiments. Error bars indicate mean ± SEM. *p≤ 0.05. **p≤ 0.01. Statistical significance was calculated using Friedman test with Dunn’s correction for multiple comparisons.

When Treg generation is fostered in coculture, polarization of other CD4+ T helper subsets may be inhibited. To determine whether VD3-liposome-primed DCs affect T cell polarization, we assessed Th1 or Th2 polarization with intracellular staining for IFN-γ (Th1 polarization) or IL-13 (Th2 polarization). Interestingly, liposome-primed moDCs significantly inhibited Th1 polarization at a concentration of 2.5 μM VD3, similar to soluble VD3-primed DCs (**Figures 5A, B**). Neither liposomes nor VD3 affected Th2 cell polarization. Hence, VD3-liposome-primed moDCs demonstrate the capacity to suppress pro-inflammatory Th1 polarization in coculture without favoring Th2 polarization. IL-17-producing CD4+ T cells (Th17 cells) are an essential hallmark of chronic inflammatory diseases, as they are involved in the pathophysiology and disease progression in several of these conditions (44–46). Accordingly, we aimed to investigate the effect of VD3-liposome primed moDCs on Th17 polarization. As stimulation by neutrophils is required for the development of Th17 cells from naïve precursors in humans (39), for these experiments, we pre-incubated DCs with soluble or DSPG liposomal VD3 and cocultured with autologous naïve CD4+ T cells and neutrophils. Strikingly, in this solid Th17-favoring coculture environment, both soluble and DSPG-VD3 liposome-primed DCs abrogated the development of Th17 cells (**Figures 5C, D**). Significant changes in Th17 induction were not seen in conditions without neutrophils (**Supplementary Figure 2**). Thus, our data suggest that liposomal VD3 treatment of DCs results in T helper polarization favoring anti-inflammatory, tolerogenic conditions.

### Priming of moDCs with liposomal VD3 distinctly changes expression of tolerogenic surface markers

**Figure 6:**
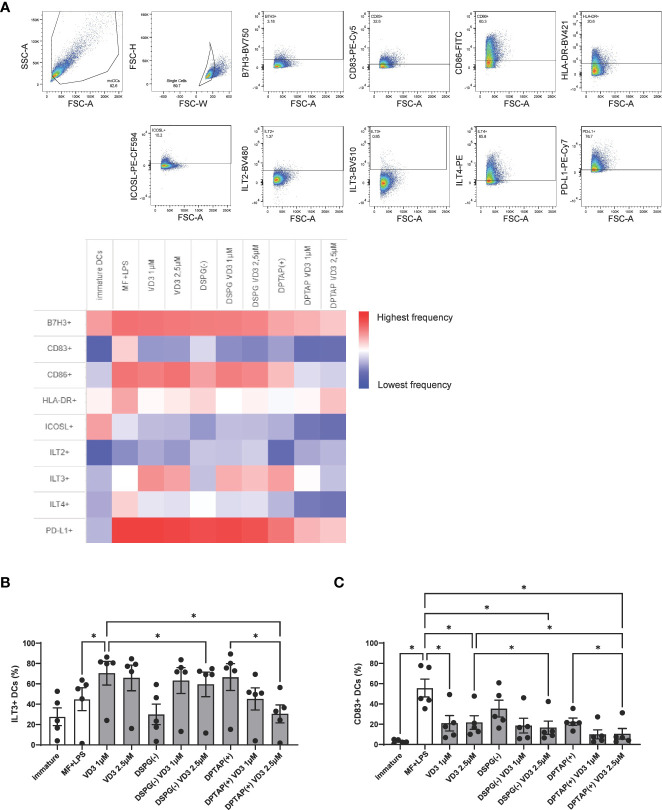
VD3-liposome treatment induces expression of ILT3 and reduces expression of CD83 on DCs. **(A)** Gating strategy and heatmap representing frequency of marker+ populations. All marker+ populations were derived from the single-cell gate. **(B)** Frequencies of ILT3 expressing DCs per condition. **(C)** Frequencies of CD83 expressing DCs per condition. Lipid concentration of empty DSPG batches shown ranges from 50-260 μg/ml and of empty DPTAP batches 14-86 μg/ml, adjusted to the lipid concentration added when using 1-2.5 μM liposome-incorporated VD3. N=5 independent experiments. Error bars indicate mean ± SEM. *p≤ 0.05. Statistical significance was calculated using one-way ANOVA with Dunnett’s correction for multiple comparisons.

To further our knowledge of the mechanism by which VD3-treated DCs can induce suppressive T cells, we phenotyped DCs after 48-hour maturation with a flow cytometry panel containing DC maturation and tolerogenic DC markers. Frequencies of marker-positive DC populations are displayed in a heatmap (**Figure 6A**). Immature DCs showed lower expression levels of most markers, compared to the rest of the activated conditions, except for the markers B7H3 and ICOSL, whereas frequencies of ICOSL+ DCs were higher in immature cells (**Figure 6A**, **Supplementary Figure 3A**). Compared to activated DCs, VD3 priming led to enhanced expression of ILT3 (**Figures 6A, B**), whereas the other tolerogenic markers examined were not significantly different in expression between activated and VD3 (liposome)-treated DCs (**Figure 6A**, **Supplementary Figures 3B-E**). Similarly, DCs activated in the presence of DSPG-loaded VD3 showed higher ILT3 expression. However, DPTAP-VD3 primed DCs displayed an opposite trend, where ILT3 expression was highest in DCs activated in presence of DPTAP empty liposomes (**Figures 6A, B**). Treatment of DCs with soluble and liposomal VD3 decreased the frequency of CD83+ cells (**Figure 6C**). Compared to activated DCs, CD86 and HLA-DR expression did not change significantly upon VD3 treatment of the cells (**Figure 6A**, **Supplementary Figures 3F, G**). Taken together, these findings suggest that DSPG-VD3 loaded and DPTAP-VD3 loaded liposomes have a differential effect on ILT3 expression of DCs, whereas all forms of VD3-priming lead to reduced expression of CD83.

### Injection of VD3-loaded DSPG or DPTAP liposomes enhances migration of CD14+ DDCs from skin biopsies

**Figure 7:**
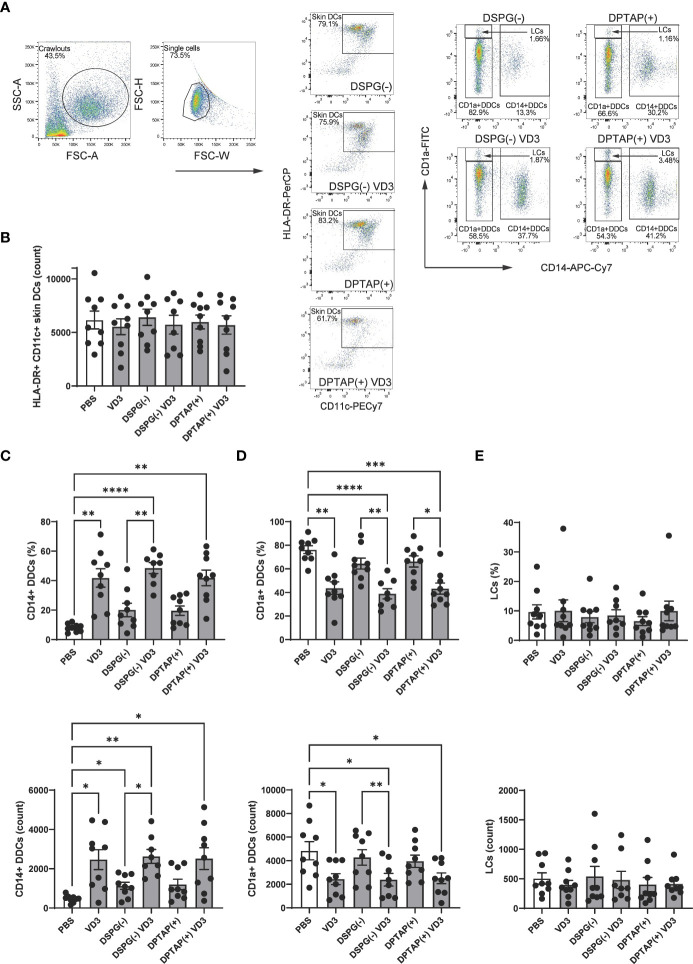
Injection of VD3-loaded DSPG or DPTAP liposomes enhances migration of CD14+ DDCs from human skin ex vivo. **(A)** Gating example for skin DCs (CD11c+ HLA-DR+ crawl-out DCs) and identification of subsets based on CD1a and CD14 staining. **(B)** Counts of CD11c+ HLA-DR+ Crawl-out DCs. **(C)** Percentages (top panel) and counts (bottom panel) of CD14a+ DDCs, **(D)** CD1a+ DCs, and **(E)** CD1a++ LCs present in crawl-out DCs, per injection condition.Soluble and liposomal VD3 concentration injected was 25 μM. Lipid concentration of empty DSPG batches ranged from 1000-2600 μg/ml and of empty DPTAP batches 860-2500 μg/ml, adjusted to the lipid concentration added when using 25 μM liposome-incorporated VD3. N=8-9 independent experiments. Error bars indicate mean ± SEM. *p≤ 0.05. **p≤ 0.01. ***p≤ 0.001. ****p≤ 0.0001. Statistical significance was calculated using mixed-effects analysis with Dunnett’s correction for multiple comparisons.

As intradermal application of liposome-loaded VD3 is an attractive mode of administering therapy, we injected 25 μM soluble VD3, anionic DPSG liposome-loaded VD3, or cationic DPTAP liposomes loaded with VD3 in *ex vivo* human skin, consistent with the approach of our previous study (40). Migratory effect of liposomal VD3 injection on (CD1a++) LCs, CD1a+ DDCs, and CD14+ DDCs was determined using flow cytometry (**Figure 7A**). The average counts of crawl-out DCs per injection condition did not differ significantly (**Figure 7B**). Compared to injection of PBS or empty liposome controls, both soluble VD3 and liposomal VD3 injection resulted in selective migration of CD14+ DDCs out of the skin biopsies (**Figure 7C**). CD14+ DDCs were present among crawl-outs in higher frequencies (**Figure 7C** **top panel**) and higher absolute counts (**Figure 7C** **bottom panel**). The observed increase in CD14+ DDC efflux mediated by VD3-liposome injections was accompanied by a decrease in percentages and counts of CD1a+ DDCs (**Figure 7D**), while percentages and counts of CD1a++ LCs remained unaltered (**Figure 7E**). Hence, we establish that liposomal VD3 injection (both loaded in anionic DSPG or cationic DPTAP liposomes) induces a similar effect on differential migration of crawl-out DCs as previously seen with soluble VD3 (40).

## Discussion

In this study, we embarked upon the development of a DC-tolerizing nanoparticulate product. We show that liposomal VD3 induces tolerogenic DCs that enhance the development of functional Tregs from naïve precursors, comparable to the effect of soluble VD3-treated DCs. We demonstrate that the T cells instructed by liposomal VD3-primed DCs have the phenotype of FoxP3+ CD127low CD25+ CD4+ T cells expressing TIGIT. Furthermore, VD3-liposome primed DCs silence Th1 and Th17-type responses. As an additional laboratory proof-of-concept, we tested the VD3-liposome formulations in an *ex vivo* human skin model and showed that they enhance migration of CD14+ DDCs from skin biopsies, similar to soluble VD3.

To our knowledge, we are the first group to add VD3-loaded liposomes to human DCs and examine the *in vitro* T cell response in detail to deliver proof-of-concept for an *in vivo* DC-targeting tolerizing therapy as a promising alternative to *ex vivo* DC vaccination. Even though we did not observe differences in tolerogenic properties of DCs treated with soluble VD3 or liposome-loaded VD3, we demonstrate comparable effects between the two treatment approaches. These findings present the *de novo* observation that VD3-loaded liposomes are suitable carriers for targeting DCs with VD3. Furthermore, we establish tolerogenic precedent for loading disease-specific antigens together with VD3 in liposomes, with the prospect of developing a non-personalized vaccine against autoimmune and allergic diseases. Additional pharmacological benefits of encapsulating VD3 in liposomes include protection from degradation and the channeling of VD3 towards APCs to establish immune tolerance without causing toxic side effects in bystander cells (10, 26).

As suppression of effector T cell proliferation is a defining hallmark of Tregs, we used a T cell suppressor assay as proof-of-concept for inducing tolerogenic DCs with VD3-loaded liposomes. Irrespective of the choice of liposome formulation, VD3-primed moDCs induced CD4+ T cells with a proliferation-suppressing regulatory capacity. This finding is strengthened by an *in vivo* study where VD3- and OVA-loaded polymer nanoparticles injected in mice significantly suppressed target cytotoxic lymphocyte proliferation, inducing OVA-specific immune tolerance (47). A recently published phase I clinical trial delivered an even stronger precedent in which liposomes containing a collagen-derived self-peptide and VD3 were subcutaneously injected in anti-citrullinated protein antibody + rheumatoid arthritis patients (48). The treatment resulted in improved disease activity, associated with an expansion of auto-antigen-specific T cells, coupled with a reduction in inflammatory myeloid cell populations and anti-citrullinated antibodies.

Upon phenotyping the Tregs resulting from VD3-DC treatment, we demonstrate a VD3-DC-induced increase in TIGIT+ FoxP3+ CD127low T cells. Furthermore, we show that the CD69+ FoxP3+ population contains higher frequencies of ICOS+ CTLA-4+ T cells. TIGIT and CTLA-4 are inhibitory receptors that corroborate the Treg identity of VD3-liposome DC-induced FoxP3+ CD127low CD25+ T cells or CD69+ FoxP3+ T cells (49, 50), while ICOS-ICOSL interactions between T cells and DCs have also been described to lead to Treg formation (51). In previous research, VD3-treatment of CD4+ T cells induced the ectonucleotidase CD39 and the T cell activation marker CD69 (44). CD39 converts extracellular ATP to immunosuppressive adenosine (42), while CD69 is emerging as a crucial protein in regulating immune responses (52). CD39, CD69, and TIM-3 enhance efficient differentiation and establishment of FoxP3+ T cells, conveying suppressive function to these cells (42, 43, 50, 53–55). Moreover, CD39 and CD69 have been linked to effective suppression of Th17 polarization (43, 44, 56). Unexpectedly, we observed a decrease in expression of these functional markers when applying VD3-treated DCs. Fast turnover, as well as intracellular instead of extracellular presence of these receptors, could be a possible explanation for these counterintuitive observations (41). However, as CD69 is also an early activation marker of memory T cells (57), decreased expression in VD3-DC treated conditions suggests that VD3-primed DCs lead to the outgrowth of less activated effector T cells. Induction of FoxP3+ T cells by VD3-DC treatment in the CD69- resting T cell population also indicates that these induced FoxP3+ cells are not activated effector T cells but Tregs.

As we observed an induction of IL-10 production of T cells stimulated with soluble or DSPG-VD3 primed DCs, which points, albeit not exclusively, to the induction of Tr1 type Tregs, we measured two important markers considered characteristic of this Treg subset, LAG-3, and CD49b (58). However, we did not succeed at reliable measurement of LAG-3 due to deficient expression levels and found no differences in expression of CD49b. Repeatedly, in the FoxP3- population of T cells, CD39 and CD69 were decreased in VD3-liposome DC conditions while expression levels of the other markers were unchanged. Hence, we could not unequivocally establish that VD3-liposome-treated DCs induced Tr1 Tregs in our cocultures.

Furthermore, we cannot exclude the possibility that VD3-treated DCs induce an altogether different Treg subset which is not fully characterized yet.

We chose two previously published formulations in this study, DSPG, and DPTAP, anionic and cationic counterparts, respectively, to load VD3. Confirming our previous results, empty DSPG or DPTAP liposomes had no tolerogenic or activating effects on moDCs, or the ensuing allogeneic T cell response and could therefore serve as internal controls in our experiments. Even though suppressive and FoxP3+ CD127low T cells were significantly induced by both DSPG-VD3 and DPTAP-VD3 treated DCs, IL-10 producing T cells were only induced by DSPG-VD3 treated DCs. In previous research, we demonstrated that DPTAP liposomes are poorly internalized by moDCs and instead adhere to the DC membrane (27). Together with the data in the current manuscript, our findings suggest that negatively charged liposomes are more suitable as delivery systems of adjuvants for future tolerance-promoting treatments.

A crucial function of Tregs is supplanting effector T cells. VD3-DCs are generally known to prevent Th1 polarization while they induce Th2 development and IL-10-producing Tregs (14, 16, 36, 40). In this study, VD3-liposome primed DCs inhibited Th1 cell development, yet they did not enhance Th2 development. This finding suggests that liposomal VD3 treatment may be preferable over soluble VD3-treated DCs in treating allergies, while autoimmune conditions may benefit from soluble and liposomal VD3.

As a disbalance between Th17 cells and Tregs is often pinpointed as pathogenetic in several inflammatory diseases (46, 59, 60), the effect of VD3-liposome treated DCs on Th17 polarization was also essential to observe. In an autologous coculture setting with neutrophils, VD3-liposome-treated DCs markedly abrogated Th17 cell development. We, and others, recently showed that DCs treated with soluble VD3 reduce Th17 development in human cell culture (37, 61, 62), adding a further argument for the suitability of VD3 for treating inflammation.

Several mechanisms of tolerance induction by VD3-treatment of DCs have been described. VD3 has been shown to downmodulate maturation of DCs, including the expression of CD83, CD86, and HLA-DR (8). Furthermore, VD3 is associated with upregulation of several surface markers considered hallmarks of tolerogenic DCs, such as ILT2, ILT3, and PD-L1 (8, 63). Together with ILT4, B7-H3, and ICOSL, these markers were demonstrated to exert tolerogenic functions *via* cognate interaction with effector T cells, rendering them anergic or transforming them into Tregs (51, 64–66). Interestingly, we only found enhanced frequencies of ILT3+ DCs upon soluble and DSPG anionic liposome VD3 treatment. Further analysis showed that only frequencies of CD83+ cells were significantly reduced by soluble or liposomal VD3-treatment of DCs, but not of HLA-DR or CD86+ cells. This contrasts with earlier studies where VD3-treatment of DCs leads to a reduction of all three markers of DC activation (8, 67). Unmeasured markers or production of a soluble factor, such as IL-10, could provide an alternative explanation for the mechanism of DC-mediated tolerance in our experiments.

When developing a DC-tolerizing vaccine, the administration route must be considered. Dermal injection of a therapeutic compound is patient-friendly, and would provide easy access to skin DCs, which can migrate to proximal lymph nodes to exert tolerogenic functions there. Hence, we have chosen an *ex vivo* skin model to investigate the suitability of the liposome carriers loaded with VD3 for a tolerogenic vaccine. Confirming previous results with soluble VD3 injection, both DSPG and DPTAP VD3-liposome injection led to selective enhancement of CD14+ DDC migration (40). In the same study, VD3-treated crawl-out skin DCs stimulated the outgrowth of suppressive Tregs, which produced less IFN-γ and contained higher frequencies of FoxP3+ cells, indicating a tolerogenic quality to CD14+ DDCs. A plausible explanation for VD3-induced CD14+ DDCs is that the other subsets present in skin start expressing CD14 upon VD3 stimulation. This explanation is supported by several studies demonstrating enhanced CD14 expression on DCs by VD3-treatment (36, 68). The DC identity and capacity of CD14+ DDCs to migrate to lymph nodes is currently under debate (69), yet CD14+ DDCs coexpressing CD141 have been described as constitutive IL-10 producers dampening skin inflammation by inducing Tregs (70). Several other studies characterized CD14+ DDCs as less immunogenic, with the capability to transform into LCs under the influence of TGF-β (71–73). Thus, the CD14+ DDC subset appears to consist of a heterogeneous mix of skin DCs, which can be molded by adjuvants, such as VD3, towards tolerogenic plasticity.

Collectively, we establish VD3-loaded liposomes as efficient therapeutic vehicles that induce DCs capable of promoting T cell tolerance *in vitro*. Together with the current findings in our study, a plethora of evidence points towards a beneficial effect of VD3 treatment on autoimmune and allergic conditions, supporting ongoing development of a DC-targeted vaccine platform using VD3 as its tolerance-promoting adjuvant.

## Data availability statement

The original contributions presented in the study are included in the article/**Supplementary Material**. Further inquiries can be directed to the corresponding author.

## Ethics statement

The studies involving human participants were reviewed and approved by the Institutional Review Board of the Amsterdam University Medical Center, per protocol nr. METC 2015_074. The patients/participants provided their written informed consent to participate in this study.

## Author contributions

NN manufactured liposomes, performed experiments, conceptualizated and validated the study, performed formal analysis and wrote the manuscript. FLV provided valuable input on liposome manufacturing and helped reviewing and editing the manuscript. RS performed experiments and manufactured liposomes. TvC performed experiments. RvR provided valuable input on data analysis and writing of the manuscript. ST provided valuable input on data analysis and writing of the manuscript. IdV provided valuable conceptual input on research methodology. TG provided valuable input on data analysis, and carefully reviewed and edited the manuscript. BS provided valuable input on liposome manufacturing, analysis of data and carefully reviewed the manuscript. EdJ conceptualized the research, provided methodology, resources and manner of data analysis for cellular assays, supervised the research, and carefully assessed the manuscript. All authors contributed to the article and approved the submitted version.
