# Supplementary material for: Liposomes loaded with vitamin D3 induce regulatory circuits in human dendritic cells
Source: Front Immunol. 2023 Jun 9;14:1137538. doi: 10.3389/fimmu.2023.1137538 (PMC10288978; doi:10.3389/fimmu.2023.1137538)
Supplement: Supplementary file 1 [file DataSheet_1.pdf]

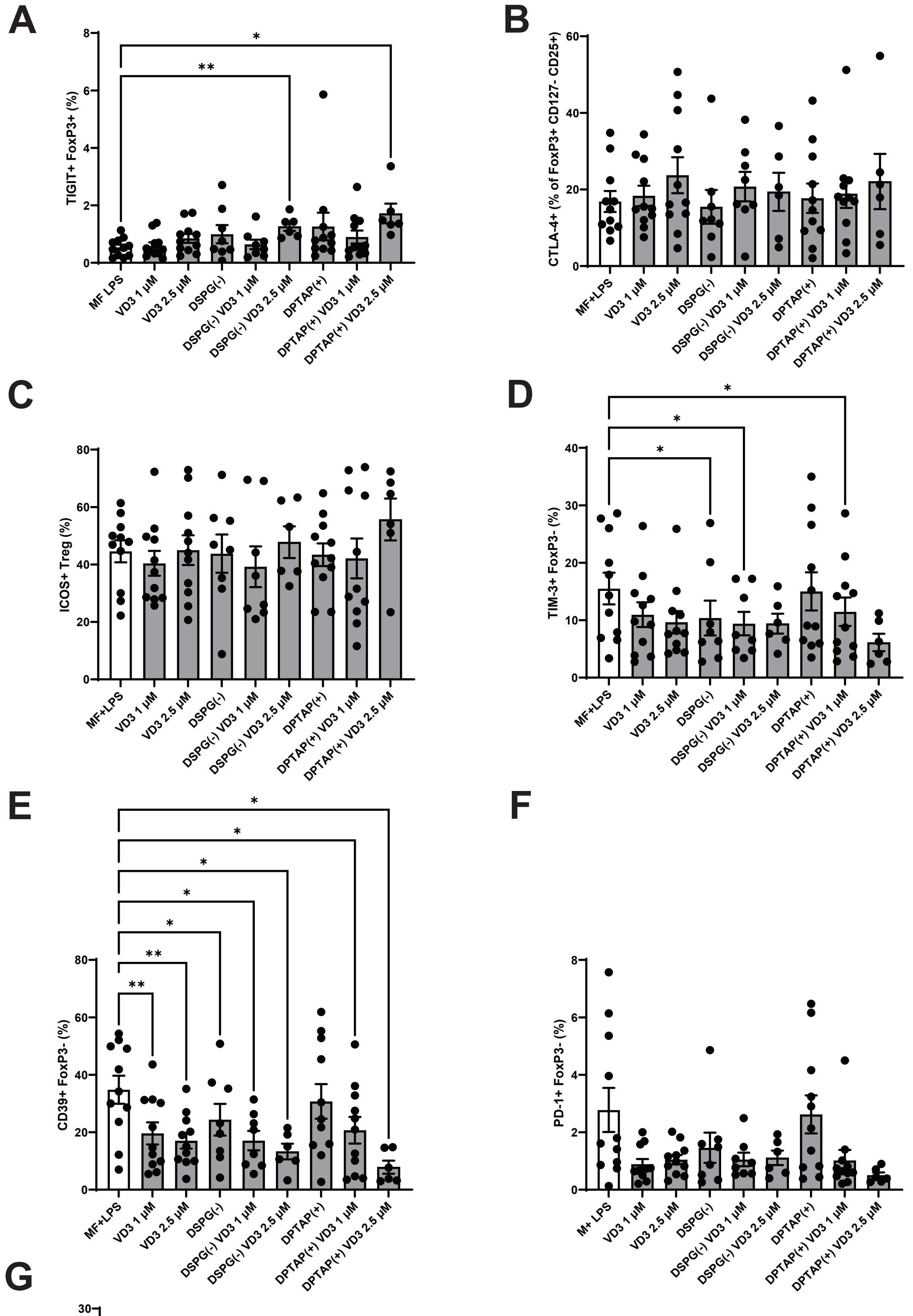

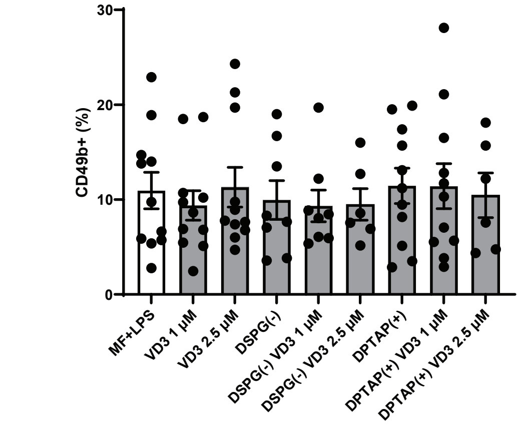

Supplementary Figure 1. VD3-liposome-treated DCs stimulate the development of CD4+ T cells with a distinct footprint of Treg markers. After 10-12 days coculture with differently primed moDCs, CD4+ T cells were stained for Treg subset and functional markers. **(A)-(G)** Frequencies of TIGIT+ FoxP3+, CTLA-4+ within FoxP3+ CD127low CD25+, ICOS+ Treg (CTLA-4+ ICOS+ FoxP3+), TIM-3+ FoxP3-, CD39+ FoxP3-, PD-1+ FoxP3-, and CD49b+ CD4+ T cells. N=5-11 independent experiments. Error bars indicate mean ± SEM. *$p \leq 0.05$. **$p \leq 0.01$. Statistical significance was calculated using mixed-effects analysis with Dunnett's correction for multiple comparisons.

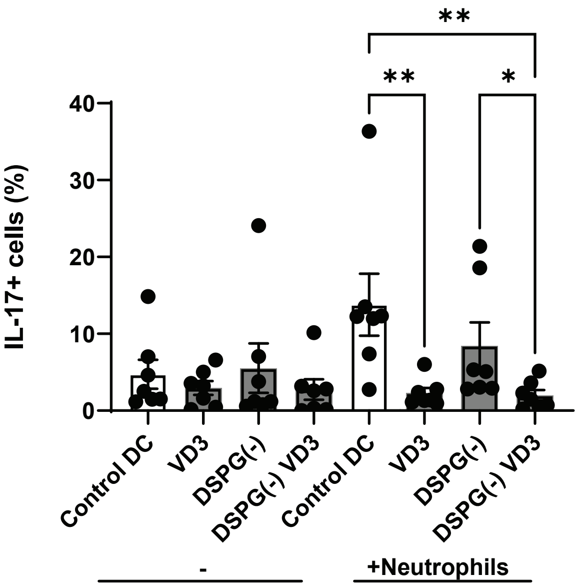

Supplementary Figure 2. Frequencies of IL-17+ CD4+ T cells after autologous coculture without or with neutrophils and differently primed moDCs. N=7 independent experiments. Error bars indicate mean ± SEM. *p≤ 0.05. **p≤ 0.01. Statistical significance was calculated using Friedman test with Dunn's correction for multiple comparisons.

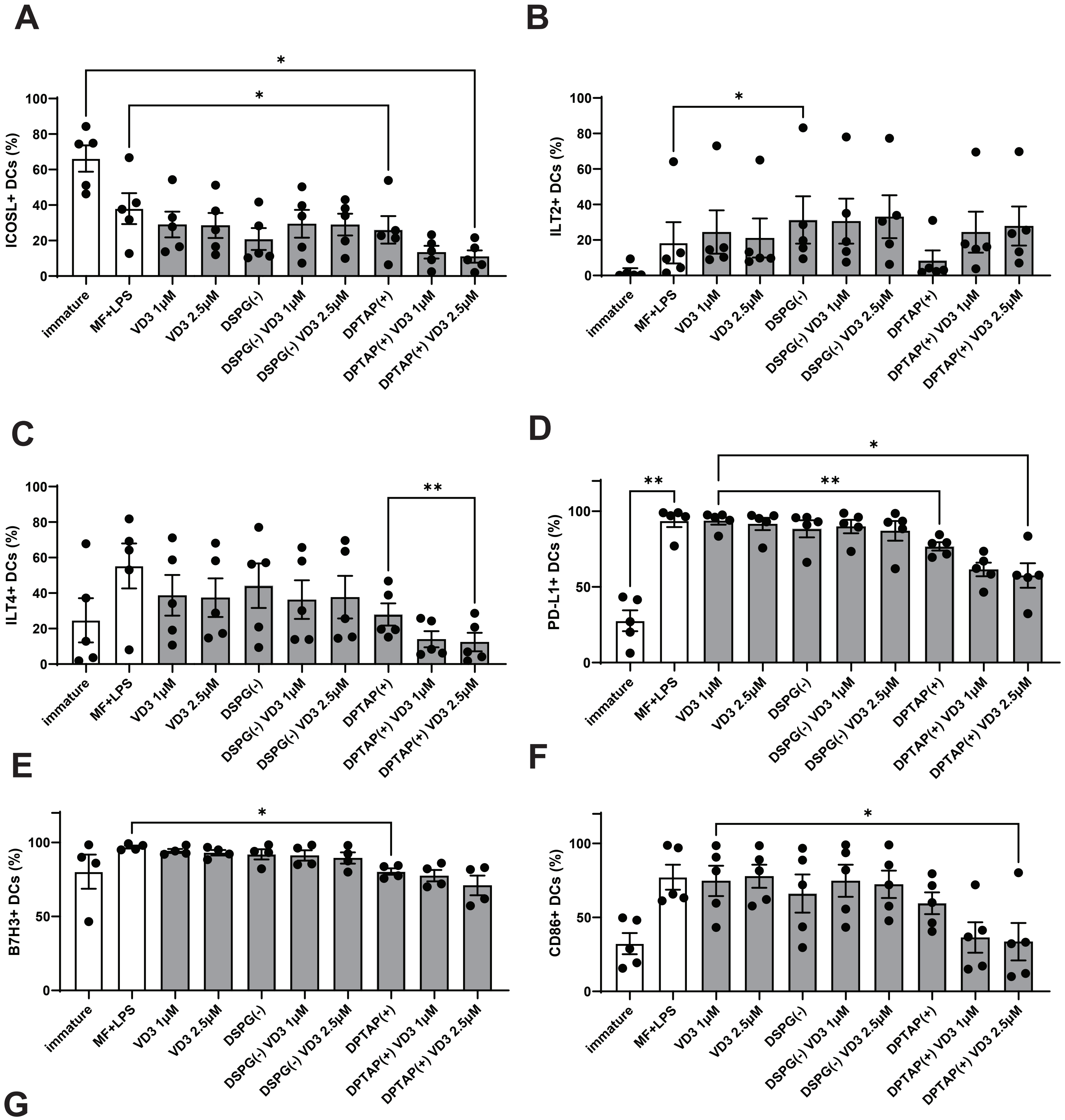

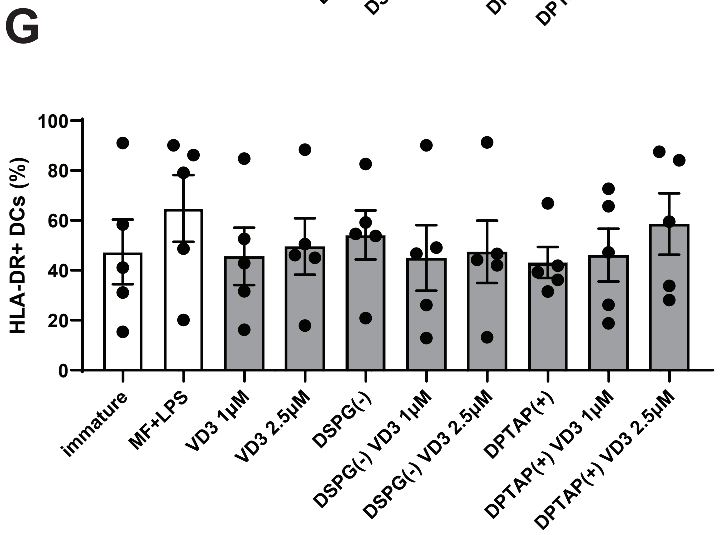

Supplementary Figure 3. Effect of VD3-liposome treatment on tolerogenic and maturation markers of DCs. (A)-(G) Frequencies of marker+ DCs are shown. N=4-5 independent experiments. Error bars indicate mean ± SEM. *p≤ 0.05. ** p≤ 0.01.
